# 

**Published:** 1922-03

**Authors:** 


### ANNOUNCEMENTS

NATIONAL DENTAL ASSOCIATION

The Twenty-sixth Annual Meeting of the above Association will be held in Los Angeles, California, July 17 to 21, 1922.

Housing Headquarters—Ambassador Hotel, Official Family; Alexandria Hotel, Delta Sigma Delta Fraternity; Clark Hotel, Psi Omega Fraternity; Hayward Hotel, Xi Psi Phi Fraternity; Registration Headquarters for all Fraternities, Ambassador.

Hotel rates, with reservation contracts may be had by addressing the Secretary or J. Elton Lang, Chairman, Com. on Halls and Hotels, 707 Auditorium Bldg., Los Angeles, California.

Railroad Fares—The fares quoted herein are as nearly accurate as can be secured until the final tariffs for 1922 are published, and are approximately correct.

The federal tax of eight per cent. on passenger and Pullman fares will be eliminated after January 1, 1922, making a substantial reduction in transportation charges, and there is a possibility of further reduction in summer fares to the Pacific Coast.

Summer tourist tickets, available for the 1922 convention, will be on sale daily, May 15, 1922, to September 30, 1922, inclusive, permitting stopover at any point en route within final return limit of October 31, 1922.

From Chicago and return to Chicago, via any direct route via Omaha, Kansas City or St. Louis, $106.80.

From Chicago and return to Chicago, via any direct line via Omaha, Kansas City, St. Louis, St. Paul or Minneapolis, $128.40. Corresponding fares from points east and south of Chicago. Variations from direct routes going and returning may be made by arrangement with local ticket agent.

| | Direct Routes | One Way via Portland |
|---|---|---|
| Cleveland, Ohio | $128.97 | $150.57 |
| Columbus, Ohio | 126.64 | 148.24 |
| Cincinnati, Ohio | 123.65 | 145.25 |
| Detroit, Michigan | 124.43 | 146.03 |
| Indianapolis, Indiana | 117.48 | 139.08 |
| New York, N. Y. | 165.61 | 187.21 |
| Pittsburgh, Pa. | 137.14 | 158.74 |

Watch for further and detailed announcements in all Dental Journals.

The Local Committee on Arrangements, per
C. M. Benbrook, *General Chairman*,
707 Auditorium Bldg., Los Angeles, California.

---

MODERN DENTISTRY FOR THE LAITY

The fourth edition of this volume is just published by the Dental Register. Alfred A. Crocker has done good work for the profession by keeping what he has termed ''odontalysis'' before the public as a matter of education for the better tooth health of the people engaged in industrial occupations. His book has helped to create greater interest in the subject, and his writing has stimulated others to engage actively in efforts to relieve suffering humanity from pain, and what is better to make it practicable for some who did not know the importance of wholesome oral health.

In addition to the new statistical materials added, a complete list of instruments and appliances makes it possible to organize for active work on the industrial basis. A complete list of corporations which have adopted dental service for their institutions give one an idea of what is being accomplished in this line. It is creditable to find that industrial corporations think it desirable to give money and spend time in such work. It ought to increase the professions respect for its own calling, and no doubt such activities will react to all concerned to make health endeavors of prime importance to the public, as well as to the dental profession. Commercial and industrial dentistry will some day work into closer alliance than we now suspect it ever has or will.

---

INDEX OF PERIODIC DENTAL LITERATURE

The second volume of this invaluable book has just been issued. This volume covers the five years of active periodic literature, 1916 to 1920. The first volume covered 1911 to 1915. Dr. Arthur D. Black and his associates have certainly done a tremendous lot of work to incorporate so much in a single volume, and the profession should give him suitable acknowledgement for what he has done. In such a work it is not practicable to make a critical review of such a tremendous undertaking, but it is certainly such a work as is much needed and will unquestionably meet with general approval, even though some errors or mistakes may have crept into the great mass of material involved. We are sure that all who have in various ways helped to assemble this material into a working basis of usefulness will rejoice in the fact that they have been permitted to do their part in so commendable and needed enterprise. The book is admirably printed and the publishers deserve credit and our thanks.

The only thing that seems now to be needed is for every member of the profession to do his part, which is small in comparison with all that has been done so well by the publishers. Every dentist and every library should possess this book, as well as the others and all future editions. This is an easy part but it is necessary that the full benefits shall result. The cost is six dollars per volume only, and certainly it will more than pay for this trifling expense. It will hundreds of times more than compensate anyone who needs only occasionally to consult it. Send money to Abram Hoffman, 381 Linwood Ave., Buffalo, New York.

WE are printing herewith a list of the Industrial Dental Clinics as they now appear in the recent fourth edition of "Modern Dentistry for the Laity." If you have any information which will help us perfect this list it will be greatly appreciated by the DENTAL REGISTER and acknowledged. The list as it now stands is the result of nearly five years' work and the result of answers to questionnaires from all sections of the United States and Canada. However, if there are any companies in your region having dental clinics which we have not listed we will be glad to receive their names so that we can add them to the list.

**Adamsville, Alabama—**

Docena Iron Works.

Tennessee Coal & Iron Co., Wenonah Mines.

Tennessee Coal & Iron Co., Ishkooda Mines.

**Acipo, Alabama—**

American Cast Iron Pipe.

**Alabama City, Alabama—**

Dwight Mfg. Co.

**Birmingham, Alabama—**
American Cast Iron Pipe Co.
Tennessee Coal & Iron Co.

**Bessemer, Alabama—**

Tennessee Coal & Iron Co., Muscoda Works.

**Chickasaw, Alabama—**
Chickasaw Shipbuilding Co.

**Ensley, Alabama—**

Tennessee Coal & Iron Co., Bayview Mines.

Westfield Clinic.

**Edgewater, Alabama—**

Edgewater Works.

**Fairfield, Alabama—**
Tennessee Coal & Iron Co.

**Johns, Alabama—**

Tennessee Coal & Iron Co.

**Davenport, California—**
Santa Cruz Portland Cement Co.

**Fresno, California—**

California Associated Raisin Growers.

**San Francisco, California—**
The Emporium.

**Denver, Colorado—**

The Colorado Fuel & Iron Co.

Daniels & Fisher Store Co.

Gates Rubber Co.

**Pueblo, Colorado—**

The Colorado Fuel & Iron Co.

**Trinidad, Colorado—**

The Colorado Fuel & Iron Co.

**New Haven, Connecticut—**
Winchester Repeating Arms Co.

**Milford, Delaware—**

The L. D. Caulk Co.

**Wilmington, Delaware—**

Dupont Powder Co.

Joseph Bancroft & Sons Co.

Electric Hose Co.

**New Haven, Connecticut—**

Kolynos Co.

L. Candee Co.

**Stamford, Connecticut—**

Yale & Towne Mfg. Co.

**South Manchester, Connecticut—**

Cheney Bros. Co.

**Atlanta, Georgia—**

Fulton Bag & Cotton Mills.

**Chicago, Illinois—**

Morris & Co.

Montgomery & Ward Co.

Armour & Co.

The Crane Co.

The Diamond Match Co.

Hart, Schaffner & Marx.

Kabo Corset Co.

Sears, Roebuck & Co.

The International Harvester Co.

**Peoria, Illinois—**

The Avery Co.

**Indiana Harbor, Indiana—**

The Inland Steel Co.

**New Orleans, Louisiana—**

D. H. Holmes Co.

Maison Blanche, Inc.

**South Brewer, Maine—**

The Eastern Mfg. Co.

**Boston, Massachusetts—**
The Hood Rubber Co.
Filene Co-operative Association.

**Watertown, Massachusetts—**
Hood Rubber Co. (Factory).

**Framingham, Massachusetts—**
The Dennison Mfg. Co.

**Chicopee Falls, Massachusetts—**

Fisk Rubber Co.

A. G. Spalding Co.

**Pittsfield, Massachusetts—**
General El. Co.

**Springfield, Massachusetts—**
Am. Bosch Magneto Co., Brightwood Station.
National Equipment Co.

**Holyoke, Massachusetts—**

Farr Alpaca Co.

**Lynn, Massachusetts—**
General Electric Co.

**Worcester, Massachusetts—**
The Norton Co.

**Wekter, Massachusetts—**

S. Slater & Sons Co.

**Wellesly Hills, Massachusetts—**
Babson Statistical Organization.

**Detroit, Michigan—**

The Ford Motor Car Co.

Solvay Process Co.

**Kalamazoo, Michigan—**

Kalamazoo Stove Co.

**Minneapolis, Minnesota—**

The Washburn-Crosby Co.

The Northwestern Knitting Co.

National Lamp Co.

Munsingwear Corporation.

**Kansas City, Missouri—**

Jones Store Co.

Montgomery & Ward.

National Lamp Works.

**St. Louis, Missouri —**

National Lamp Works.

Union Electric Light & Power Co.

Monsanto Chemical Works.

Stix, Baer & Fuller Dry Goods Co.

Century Electric Co.

B. Nugent & Bros. Dry Goods Co.

**Concord, New Hampshire—**
Union District Dental Clinic.

**Manchester, New Hampshire—**
The Amoskeag Co.

**Nashua, New Hampshire—**

Nashua, City Dental Clinic.

Maginnis Cotton Mills.

**Harrison, New Jersey—**

Harrison Lamp Works.

The General Electric Co.

**Hurley, New Mexico—**

Chino Copper Co.

**Brooklyn, New York—**

Schrader & Sons.

**Buffalo, New York—**
Larkin Co.
American Radiator Co.
Buffalo Miniature Lamp Works.
Pierce-Arrow Motor Car Co.

**Johnson City, New York—**
Endicott-Johnson Co.
E.-J. Workers Medical & Relief.

**McGregor, New York—**
Metropolitan Life Insurance Co.

**New York City, New York—**

International Garment Workers' Union.

Lord & Taylor.

Macy Mutual Aid Assn.

James McCreery & Co.

Metropolitan Life Insurance Co.

New York Telephone Co.

John Wanamaker Store.

Colgate & Co.

Sanatorium.

**Niagara Falls, New York—**

Oldsbury Electro Chem. Co.

**Rochester, New York—**
The Bousch & Lomb Co.

**Schenectady, New York—**

General Electric Co.

**Akron, Ohio—**

The Firestone Tire & Rubber Co.

The B. F. Goodrich Co.

**Cincinnati, Ohio—**

The Cincinnati Milling Machine Co.

The R. K. LeBlond Machine Tool Co.

The Worthington Pump Co.

The Lunkenheimer Co.

**Cleveland, Ohio—**

The Bailey Co.

The Joseph & Feiss Co.

The Kaynes Co.

National Lamp Works of General El. Co. (Parent Co. of all Natl. Lamp Works.)

H. & E. Blouse Co.

**Columbus, Ohio—**

Jeffries Mfg. Co.

**Dayton, Ohio—**
Dayton Electric Laboratories Co.
The Dayton Metal Products Co.
The National Cash Register Co.
Domestic Engineering Co.

**Hamilton, Ohio—**

The Hooven-Owens-Rentschler Co.

**Newcomerstown, Ohio—**

J. B. Clow Co.

**Warren, Ohio—**
National Lamp Works of General El. Co.

**Youngstown, Ohio—**
National Lamp Works of General El. Co.

**Erie, Pennsylvania—**

Erie Forge Co.

**Philadelphia, Pennsylvania—**

Powers, Weightman, Rosengarten Co.

John Wanamaker.

The S. S. White Dental Mfg. Co.

Lit Brothers.

J. G. Brill Co.

John B. Stetson Co.

The American Pulley Co.

**Pittsburgh, Pennsylvania—**

The Armstrong Cork Co.

The H. J. Heinz Co.

Lee S. Smith & Son Co.

Kaufmann's, The Big Store.

**Reading, Pennsylvania—**

Berkshire Knitting Mills.

Berkshire Knitting Machine Co.

The Narrow Fabric Co.

**Bristol, Rhode Island—**

National India Rubber Co.

**Central Falls, Rhode Island—**
Rhode Glass Division (Natl. Lamp Works, General Electric Co.).

**Esmond, Rhode Island—**

Esmond Mills.

**Bosevaine, Virginia—**

Pocahontas Fuel Co.

**Itman, West Virginia—**

Pocahontas Fuel Co.

**Jenkinjones, West Virginia—**

Pocahontas Fuel Co.

**Maybeury, West Virginia—**

Pocahontas Fuel Co.

**War, West Virginia—**

Williams Pocohontas Coal Co.

**Appleton, Wisconsin—**

Kimberly-Clark Co.

**Kohler, Wisconsin—**

Kohler Mfg. Co.

**Milwaukee, Wisconsin—**

Phoenix Knitting Mills.

Cutler-Hammer Mfg. Co.

Chain Belt Co.

Wisconsin Bridge Works.

**Neenah, Wisconsin—**

Kimberly-Clark Co.

**Niagara, Wisconsin—**

Kimberly-Clark Co.

**Toronto, Canada—**

T. Eaton Co., Ltd.

